# Supplementary material for: LncRNA-Mediated Tissue-Specific Plastic Responses to Salinity Changes in Oysters
Source: Int J Mol Sci. 2025 May 9;26(10):4523. doi: 10.3390/ijms26104523 (PMC12111029; doi:10.3390/ijms26104523)
Supplement: Supplementary file 1 [file ijms-26-04523-s001.zip › ijms-3568521-Supplementary figure.pdf]

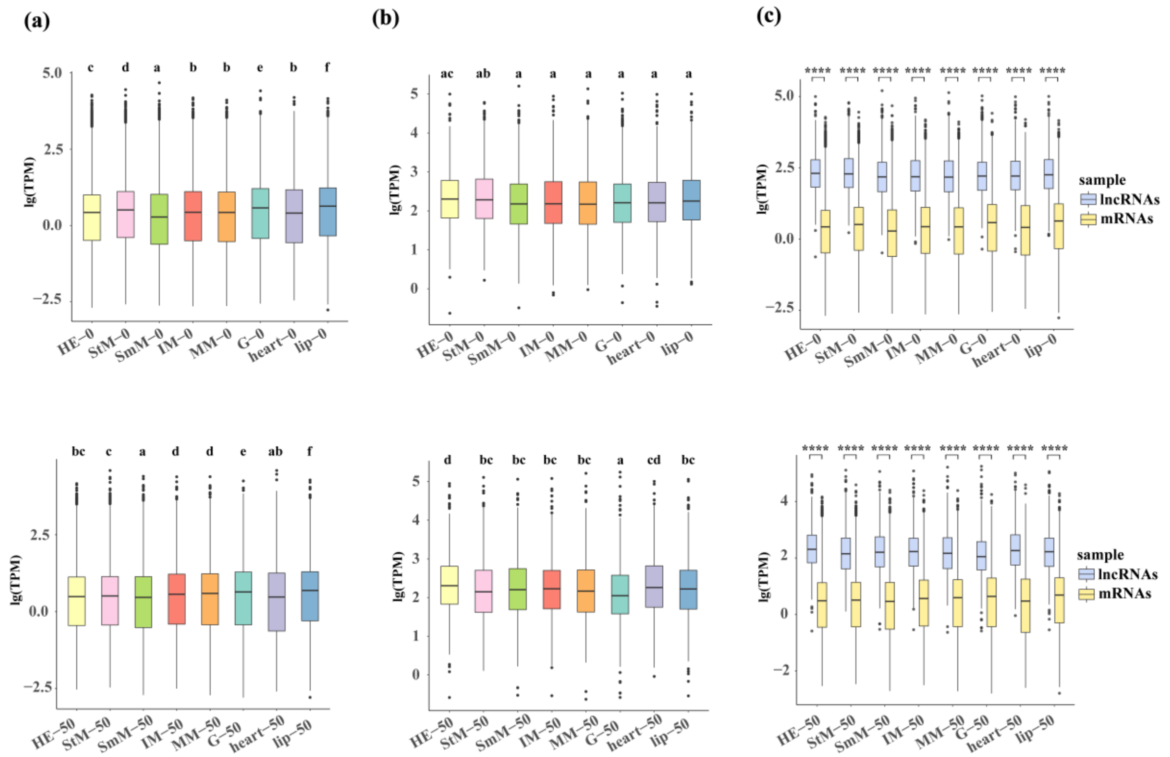

Figure S1. Genome-wide expression levels of mRNAs (a), lncRNAs (b), and their comparison (c) under hypo- (0‰) and hyper- (50‰) saline conditions. Letters and asterisks denote significant differences ($p < 0.05$).

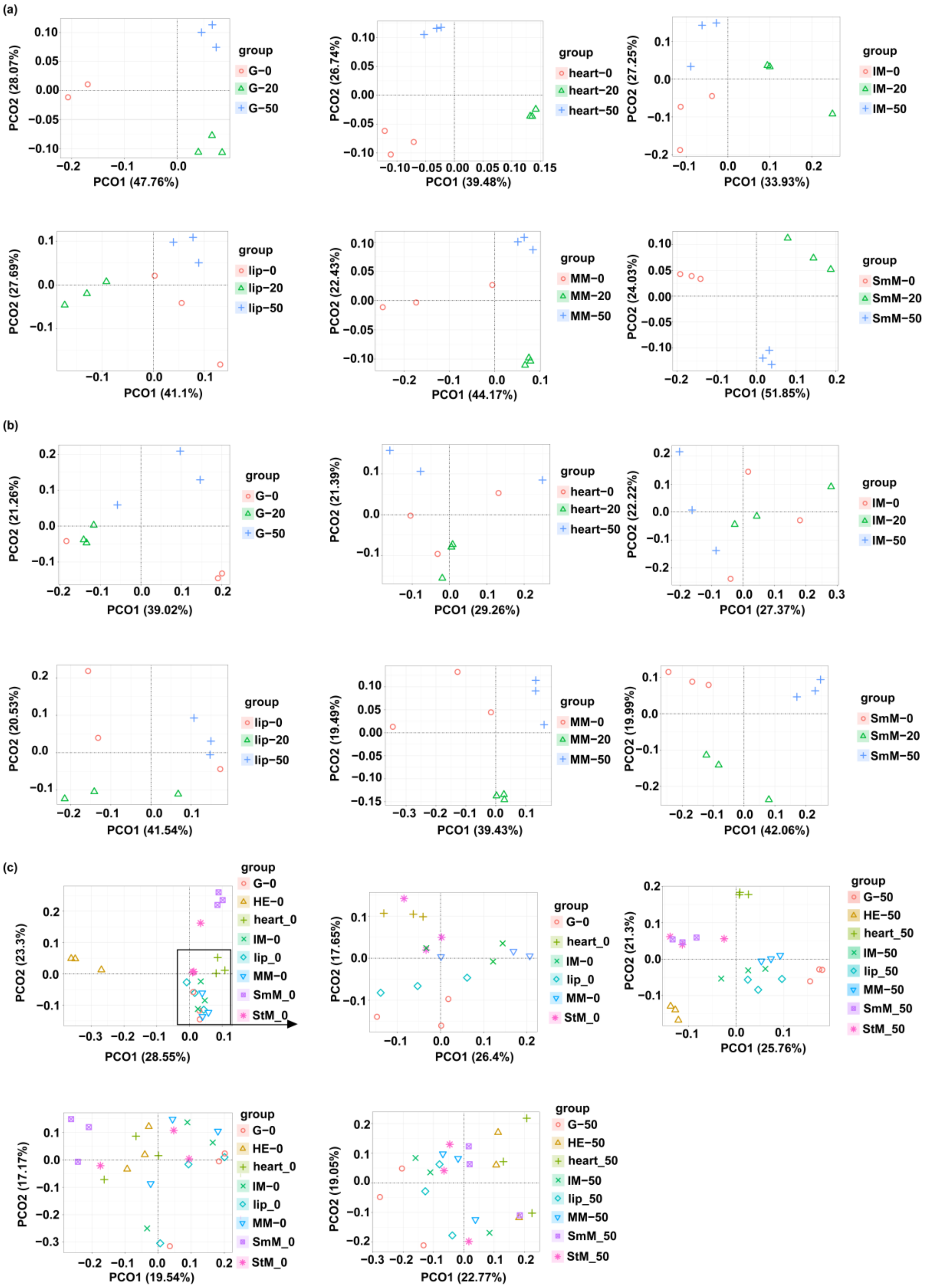

Figure S2. PCoA clustering of genome-wide expression profiles for mRNAs and lncRNAs. (a) Clustering of mRNAs in six tissues (lip, G, IM, MM, heart, SmM) under three salinity

conditions. (b) Clustering of lncRNAs in six tissues (lip, G, IM, MM, heart, SmM) under three salinity conditions. (c) Clustering of mRNAs and lncRNAs across eight tissues under hypo- (0‰) and hyper- (50‰) saline conditions.

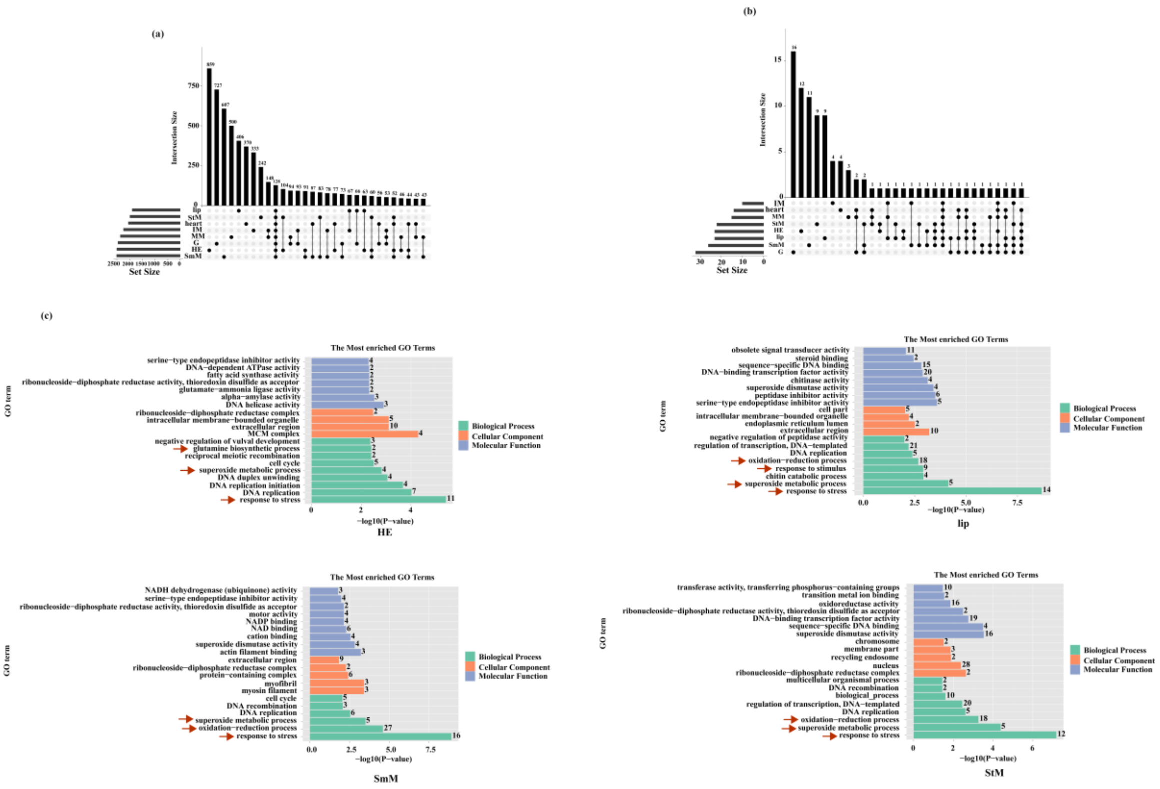

Figure S3. The number of DEGs (a) and DELRs (b) across eight tissues under salinity stresses. (c) Enrichment analysis of DEGs in response to salinity in HE, lip, SmM and StM tissues.

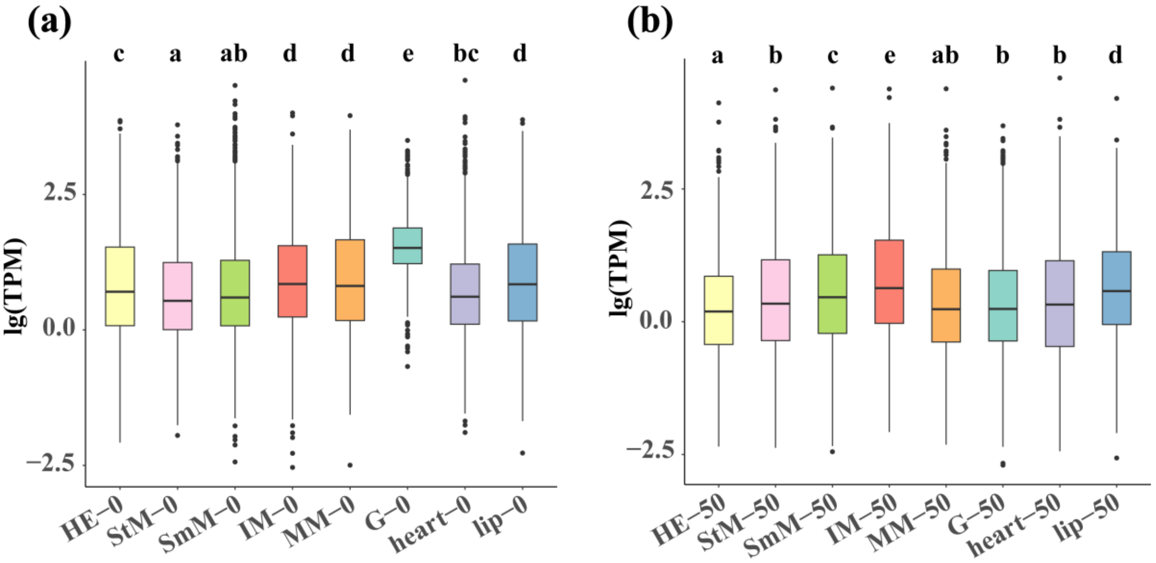

Figure S4. Expression levels of DEGs under hypo- (0‰) and hyper- (50‰) saline stresses across eight tissues. Letters indicate significant differences ($p < 0.05$).

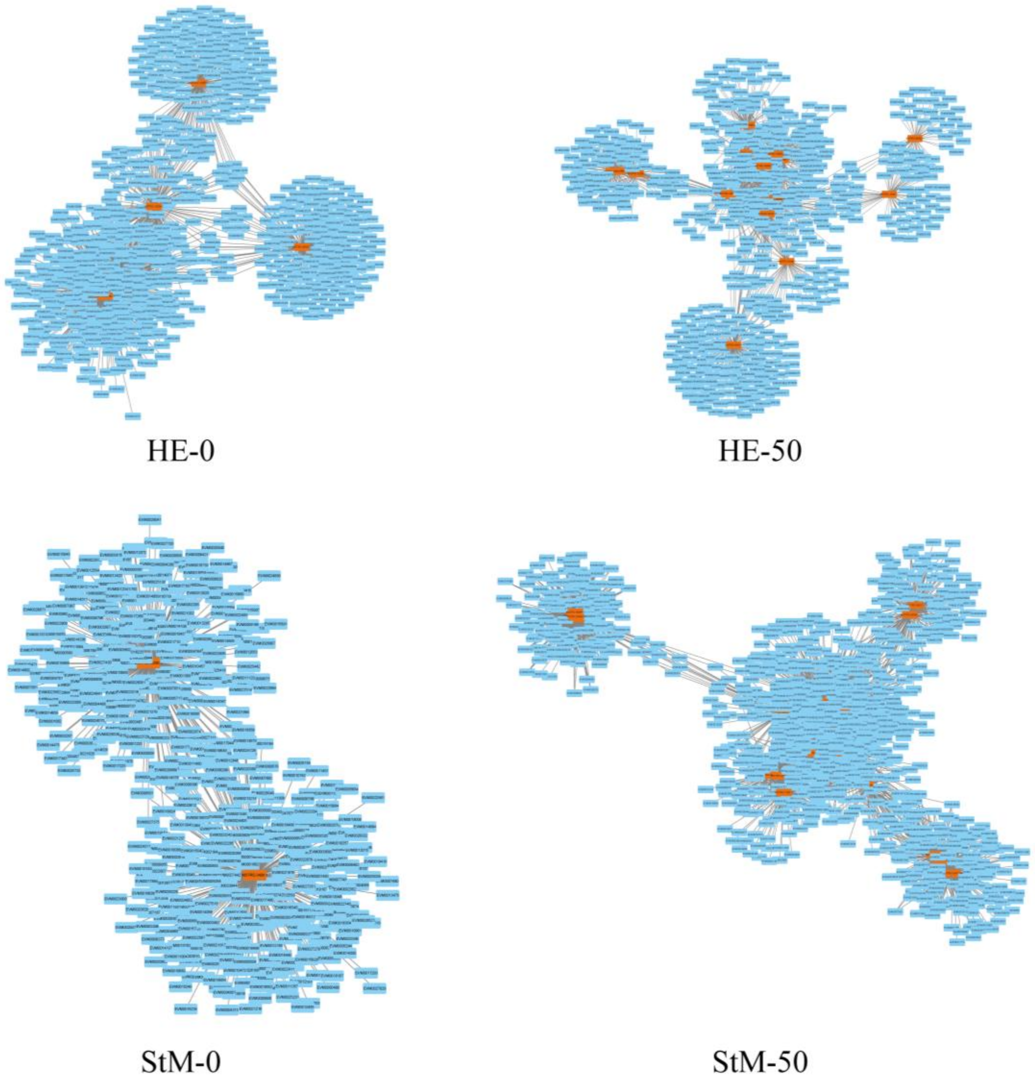

Figure S5. The network of DELRs and their correlated DEGs (scL-DEGs) in HE and StM under hypo- (0‰) and hyper- (50‰) saline conditions. Orange nodes represent DELRs, while blue nodes represent scL-DEGs.

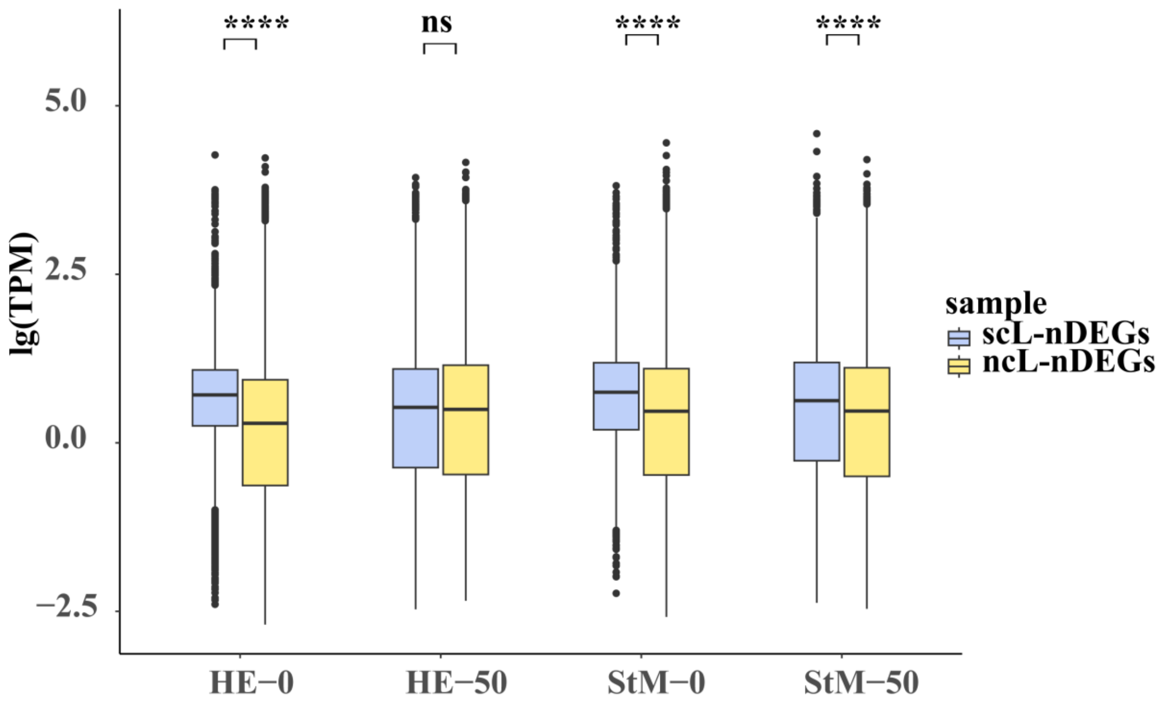

Figure S6. Expression levels of scL-nDEGs and ncL-nDEGs in HE and StM under hypo- (0‰) and hyper- (50‰) saline conditions. Asterisks denote significant differences ($p < 0.05$).

Table S1. Summary of RNA-seq data.

Sample	Clean bases	Clean reads	Mapped Reads	Mapping rate (%)	Q30 (%)	GC (%)
lip-0-1	6,962,122,878	47,705,934	30,610,425	64.16	95.08	43.34
lip-0-2	6,917,492,202	47,318,134	30,905,971	65.32	95.75	43.63
lip-0-3	6,942,906,137	48,141,776	31,030,340	64.46	96.20	42.52
lip-20-1	6,948,831,103	47,976,898	30,534,720	63.64	95.30	43.14
lip-20-2	6,945,828,784	48,942,418	30,237,715	61.78	96.57	42.92
lip-20-3	6,942,906,000	48,141,776	29,392,787	61.05	96.00	43.00
lip-50-1	6,971,339,119	48,199,882	30,968,120	64.25	96.06	43.65
lip-50-2	6,915,184,917	47,991,708	31,353,331	65.33	95.91	43.55
lip-50-3	6,930,870,971	48,494,694	32,771,217	67.58	94.93	44.04
HE-0-1	6,619,127,618	45,333,750	29,850,813	65.85	95.70	44.55
HE-0-2	7,036,263,160	48,344,380	30,139,044	62.34	94.79	43.67
HE-0-3	6,913,220,275	48,955,982	30,381,656	62.06	96.29	43.57
HE-20-1	6,988,486,716	48,004,222	30,948,679	64.47	95.97	43.39
HE-20-2	7,021,410,372	48,162,274	29,883,305	62.05	94.65	42.79
HE-20-3	7,064,989,601	48,788,896	30,792,976	63.11	96.31	44.07
HE-50-1	6,973,468,793	47,721,214	32,108,646	67.28	96.12	44.05
HE-50-2	7,063,383,428	48,441,720	32,460,953	67.01	94.61	44.00
HE-50-3	6,940,873,650	47,564,510	29,270,959	61.54	96.10	43.09
StM-0-1	7,009,698,671	48,116,052	30,893,253	64.21	94.74	43.79
StM-0-2	6,488,981,974	44,368,254	24,839,239	55.98	95.71	42.64
StM-0-3	7,031,560,806	47,914,688	28,734,838	59.97	95.63	42.79
StM-20-1	6,946,400,060	48,085,662	29,876,736	62.13	96.12	43.02
StM-20-2	7,024,498,736	48,538,580	30,933,442	63.73	96.38	43.27
StM-20-3	6,937,379,641	47,711,850	29,605,039	62.05	96.09	43.13
StM-50-1	6,921,275,158	47,546,158	30,336,278	63.80	95.18	43.19
StM-50-2	7,072,895,149	48,492,948	30,469,593	62.83	96.19	43.04
StM-50-3	7,068,468,002	48,472,460	30,829,745	63.60	96.25	43.18
SmM-0-1	6,949,493,966	47,514,316	30,451,809	64.09	95.42	44.17
SmM-0-2	7,047,528,067	48,296,742	30,803,592	63.78	95.01	44.02
SmM-0-3	6,892,551,049	47,272,734	29,353,042	62.09	95.68	43.79
SmM-20-1	7,039,395,495	48,742,924	31,530,117	64.69	96.38	43.50
SmM-20-2	6,904,748,293	47,801,394	29,593,713	61.91	96.27	42.80
SmM-20-3	7,005,111,796	48,514,522	31,079,117	64.06	96.45	43.93
SmM-50-1	6,994,031,204	47,935,648	31,202,017	65.09	95.65	43.99
SmM-50-2	6,931,978,098	47,768,148	30,743,825	64.36	95.08	43.80
SmM-50-3	6,873,271,566	47,351,874	28,539,400	60.27	95.64	42.77
G-0-1	6,907,745,149	47,127,390	30,327,455	64.35	95.48	43.44
G-0-2	7,033,165,689	48,213,338	31,743,060	65.84	95.63	43.82
G-0-3	6,999,103,663	47,595,676	31,693,276	66.59	95.31	43.69
G-20-1	7,024,395,672	48,365,516	31,178,319	64.46	96.28	43.75
G-20-2	7,062,684,643	49,103,236	30,288,562	61.68	96.27	43.00
G-20-3	6,922,469,554	47,727,780	30,138,334	63.15	96.04	43.40
G-50-1	6,960,713,274	48,607,686	32,356,145	66.57	95.37	43.90

G-50-2	6,977,106,858	48,179,122	31,925,269	66.26	95.72	43.70
G-50-3	7,020,875,568	48,218,856	32,100,971	66.57	95.27	43.48
MM-0-1	6,976,696,323	47,793,498	29,387,046	61.49	95.58	43.49
MM-0-2	7,023,519,080	47,948,942	30,275,664	63.14	95.03	44.21
MM-0-3	6,927,901,350	47,428, 420	30,299,038	63.88	93.69	43.31
MM-20-1	6,942,039,107	48,008,392	31,015,544	64.60	96.24	43.50
MM-20-2	7,014,943,373	48,368,082	31,850,079	65.85	96.23	43.66
MM-20-3	6,927,390,925	48,140,100	30,649,889	63.67	94.93	43.35
MM-50-1	6,866,731,522	46,942,522	31,154,189	66.37	95.36	43.66
MM-50-2	7,055,787,712	48,394,932	31,670,586	65.44	94.74	43.36
MM-50-3	6,904,214,170	47,288,074	31,838,666	67.33	94.68	43.84
IM-0-1	7,003,910,641	47,902,184	32,216,550	67.25	95.79	43.97
IM-0-2	6,993,246,336	48,321,260	31,772,236	65.75	95.69	44.04
IM-0-3	6,987,831,371	48,807,824	30,923,614	63.36	95.57	43.63
IM-20-1	6,412,877,524	44,085,078	27,798,002	63.06	96.32	43.02
IM-20-2	6,864,777,760	47,606,360	31,119,602	65.37	96.10	43.58
IM-20-3	7,014,707,771	48,279,924	29,567,708	61.24	96.40	42.40
IM-50-1	6,935,399,501	47,814,378	31,040,310	64.92	95.91	43.12
IM-50-2	7,017,338,518	48,415,248	31,305,097	64.66	94.93	43.14
IM-50-3	7,021,923,725	48,110,356	31,438,887	65.35	95.42	43.46
heart-0-1	6,822,814,872	47,405,034	30,291,477	63.90	95.87	43.65
heart-0-2	6,674,706,232	46,264,148	28,721,448	62.08	96.20	43.44
heart-0-3	6,900,930,052	47,277,234	28,084,112	59.40	95.45	43.36
heart-20-1	6,905,357,348	48,026,634	32,387,470	67.44	96.19	44.68
heart-20-2	6,900,561,498	47,732,656	31,070,262	65.09	96.41	43.94
heart-20-3	7,038,528,049	48,983,994	30,989,534	63.26	96.02	43.69
heart-50-1	7,063,716,078	49,124,328	33,507,119	68.21	96.22	44.07
heart-50-2	6,897,662,249	47,816,574	32,269,909	67.49	96.46	44.15
heart-50-3	6,949,971,685	48,160,492	30,812,950	63.98	95.96	43.65
**Total**	500,191,718,297	3,400,831,970	2,208,664,831	/	/	/
**Average**	6,947,107,199	47,899,042	30,675,900	64.04	95.71	43.52
**Min**	6,412,877,524	44,085,078	24,839,239	55.98	93.69	44.68
**Max**	7,072,895,149	49,124,328	33,507,119	68.21	96.57	42.40

Note: Gill [G], smooth muscle [SmM], striated muscle [StM], marginal mantle [MM], inside mantle [IM] and hepatopancreas [HE].
